# Evaluation of METase-pemetrexed-loaded PEG–PLGA nanoparticles modified with anti-CD133–scFV for treatment of gastric carcinoma

**DOI:** 10.1042/BSR20171001

**Published:** 2018-01-30

**Authors:** Lin Xin, Hou-Ting Zhang, Wei-Feng Yang, Yi-Fan Li, Chuan Liu

**Affiliations:** Department of General Surgery, The Second Affiliated Hospital of Nanchang University, Nanchang, Jiangxi 330006, China

**Keywords:** CD133, gastric carcinoma, methioninase, pemetrexed, PEG-PLGA nanoparticles, scFV

## Abstract

PEG–PLGA nanoparticles (NPs) modified with anti-CD133 and tumor-targeting single-chain antibody fragment (scFV–NPs) for systemic delivery of methioninase (METase) and pemetrexed for gastric carcinoma were successfully formulated. The structure characterization and biological functions of METase-pemetrexed-loaded scFV–PEG–PLGA NPs (scFV–METase/pemetrexed–NPs) *in vitro* were investigated. Functional scFV–PEG–PLGA NPs or PEG–PLGA NPs present low cell cytoxicity in CD133+ SGC7901 cells. scFV–METase/pemetrexed–NPs (scFv–M/P–NP) was more effective in inhibiting tumor growth (including cell growth and migration ability) in CD133 positive expressed gastric cancer cells than METase/pemetrexed-NPs (M/P–NP). Moreover, METase enhanced the inhibitory effect of pemetrexed on thymidylate synthase (TS) synthesis and cell apoptosis. We have demonstrated the application of scFV-targeted PEG–PLGA NPs as a new potential strategy to enhance treatment benefits for gastric carcinoma.

## Introduction

Gastric carcinoma is the second leading cause of cancer death worldwide [1]. The prevalence of gastric carcinoma in China is among the highest in the world, along with Japan and Korea [2]. At present, the pathogenesis of gastric carcinoma remains unclear, and the prospect for patients with gastric carcinoma is not optimistic. Therefore, it becomes a research focus to explore new early diagnostic tools and therapeutic techniques. CD133 is a pentaspan transmembrane glycoprotein, with a molecular weight of 120 kDa [3]. CD133 is highly expressed in various solid tumors, including colon cancer [4] and glioblastoma [5]. Recently, it has been reported that a moderate to high percentage of gastric cancer tumor samples have CD133 expression with moderate to strong membranous and apical expression [6], which identifying that CD133 could be acted as a potential therapeutic target for antibody–drug conjugates in gastric carcinoma, and raising the possibility of molecular targeting therapy in the most aggressive malignancy.

In recent years, anticancer-drug-loaded nanoparticles (NPs) and microparticle drug delivery systems have been extensively utilized and have become an important research area in various cancer therapy [7]. Application of the biodegradable polymer poly(lactic-co-glycolic) acid has shown immense potential as a drug delivery carrier [8]. The poly(D,L-lactide-co-glycolide acid) (PLGA) coated polyethylene glycol possesses diverse advantages including high biocompatibility, biodegradability, and low toxicity [9], and has been widely used for intravenous administration of therapeutic agents. Insertion of polyethylene glycol (PEG) into the polymeric structure, as block copolymers, is a simple way to spawn various formulations, such as NPs, hydrogels, and injectable systems possessing more favorable pharmacokinetic parameters [10]. Conjugation or encapsulation of therapeutic proteins with PEG or PEG modified NPs has also been shown to produce significant advantages, especially in the aspect of reducing antigenicity [11]. Moreover, block copolymers formed by PLGA combination with PEG have been proven to promote drug loading capacity, and have been widely used as drug carrier materials [12].

Methioninase (METase), a powerful enzyme drug to methionine depletion, has been widely used as a therapeutic strategy for gastric carcinoma in clinic [13]. The up-regulated methionine requirement for cell growth has presented in the great mass of cancer cells, as compared with the normal cells. Moreover, methionine starvation therapy using a methioninase-free diet or total parenteral nutrition (TPN) prolongs the survival time of high-stage gastric carcinoma patients [14]. Pemetrexed is a newly developed multitargeted antifolate enzyme inhibitor, which has promising clinical activity against a variety of tumors, including gastric carcinoma [15,16]. Wei et al. [17] found that pemetrexed based chemotherapy is mildly effective in treating patients with metastatic gastric cancer with tolerable toxicity. Liu et al. [18] shown that treatment with pemetrexed based chemotherapy is active and is well tolerated in patients with advanced gastric cancer. However, the effects of coencapsulation of METase and pemetrexed NPs on gastric carcinoma cells remain unknown.

Single-chain variable fragment (scFv) is included in tumor-targeting human monoclonal antibodies, and is used to increase the targeting effect and promotes the effective delivery of cancer therapy drugs due to its high affinity and low antigenicity [19]. The objective of the current research is to evaluate the biological functions of METase-pemetrexed-loaded PEG–PLGA NPs modified with anti-CD133–scFV on SGC 7901 in the treatment of gastric carcinoma.

## Materials and methods

### Preparation of PEG–PLGA polymer

The process of activated polymer was as follows: lactic acid (LA) and glycolic acid (GA) were mixed with the ratio of 50:50 (MW = 34 kDa) to form PLGA polymer. Polymer was dissolved in dichloromethane (DCM) and the mole ratio of PLGA:1-ethyl-3-(3-dimethylaminopropyl) carbodiimide (EDC):*N*-hydroxy succinimide (NHS) = 1:10:10 under nitrogen environment for 24 h at room temperature. The solvent was evaporated in vacuum, and the residual solution was added to ether to obtain precipitation after centrifugation (15000 × ***g***, 10 min, 4°C), then vacuum drying was performed and the activated PLGA–NHS was obtained. The system and PEG were mixed in 1:1 mole proportion at the room temperature in nitrogen condition for 24 h. The solvent was evaporated in vacuum, and the residual solution was added to methanol to obtain precipitation after centrifugation (15000 × ***g***, 10 min, 4°C), then vacuum drying was performed and the activated PEG–PLGA was acquired [20].

### Preparation of various NPs

The modified, double emulsion, solvent evaporation method (W1/O/W2 dual emulsion-solvent evaporation method) used in this work was based on the previous study [21]. pcDNA3.1-METase was synthesized by Shionogi Co., Ltd. (Osaka, Japan). Pemetrexed and pcDNA3.1-METase were dissolved in PBS/Tween20 solution to form the internal aqueous phase (W1). PEG–PLGA polymer was mixed with DCM to form the oil phase (O). PF68 solution was acted as external aqueous phase (W2). The first emulsion (W1/O) was injected directly into 50 ml of polyvinyl alcohol (PVA) solution under agitation and emulsification continued at 10000 rpm for 6 min to produce a W1/O/W2 emulsion. The system was stirred under vacuum to evaporate DCM and prevent pore formation on the surface of NPs, then these NPs were centrifuged at 10000 rpm for 30 min, washed with distilled water and 2% w/v sucrose solution, and freeze dried. A single emulsion solvent evaporation (W/O) method was used to obtain PEG–PLGA NPs (also referred to NPs). At last, all final products were stored in a desiccator at room temperature. The specific compositions of NPs were presented in Table 1.

**Table 1:** The specific composition of various NPs

Nanoparticle	NPs (PEG-PLGA)	Pemetrexed-NPs (PEG-PLGA-pemetrexed)	METase/Pemetrexed-NPs (PEG-PLGA-METase-pemetrexed)
W1		PBS/Tween (8:2) Pemetrexed	PBS/Tween (8:2) pcDNA-ME Tase Pemetrexed
O	DCM	DCM	DCM
	PEG-PLGA polymer	PEG-PLGA polymer	PEG-PLGA polymer
W2	3% PF68 (w/v)	3% PF68 (w/v)	3% PF68 (w/v)

W1 means internal aqueous phase, O means oil phase, W2 means external aqueous phase, and DCM means dichloromethane.

### Synthesis of anti-CD133–scFV modified various NPs

The above-mentioned NPs and anti-CD133–scFV were dissolved in acetone. The mixtures were poured into Millipore water solution with solvent:water = 1:5. NPs were formed and gently stirred at room temperature for 4–5 s to evaporate organic solvent. NPs were coincubated with EDC and NHS for 15 min at room temperature with gentle stirring. The activated particles were covalently linked to scFv at room temperature and vortexed. The anti-CD133–NPs conjugated with scFv were purified from unconjugated proteins by ultrafiltration. The average size of NPs derivatives was analyzed by dynamic light scattering (DLS). The zeta potential of NPs was evaluated in deionized water solution. Transmission electron microscopy (TEM) system was used to determine the shape and surface morphology of NPs produced.

### Release profile

The release profiles of METase from METase/Pemetrexed–NPs with or without scFV decoration were determined in accordance with our previous study [22]. The release profiles of Pemetrexed were assessed as follows: coencapsulated METase and pemetrexed NPs were added into a dialysis bag with phosphate buffer solution (PBS) in 1:19 volume ratio. The dialysis bags were then put into the test tube with 20.0 ml of PBS and incubated at 37°C and gently rotated. At predetermined time points, NPs were collected by centrifugation, and supernatant was used to quantify the released Pemetrexed by spectrophotometer.

### Cell culture

The human GCC lines SGC7901 and MKN45 were obtained from American Type Culture Collection (ATCC, Manassas, VA, U.S.A.). SGC7901 and MKN45 cells were cultured in RPMI-1640 (Gibco, Grand Island, NY, U.S.A.) containing 10% FBS at 37°C and 5% CO_2_.

### *In vitro* growth inhibition of CD133+ SGC-7901 cells

CD133+ SGC7901 and MKN45 gastric cancer cells were isolated by magnetic-activated cell sorting (MACS) method. They were seeded into 96-well black plates at a density of 5000–10000 cells/well, and incubated for 24 h at 37°C and 5% CO_2_. Then, cells were treated with different kinds of NPs, and untreated cells were used as controls. Cell viability was estimated by MTT assay.

### Cell migration assay

A 24-well insert with an 8-mm pore size was employed for the CD133+ marked SGC-7901 and MKN45 cell migration analysis. Briefly, the cells were dissociated with Accutase, resuspended in 100 μl of serum-free medium, and placed in the upper chamber (without or precoated with 500 ng/ml Matrigel solution for the migration assay), while 600 μl of 10% FBS medium was placed in the lower chamber. After incubation at 37°C for 48 h, the cells on the upper membrane surface were scraped off. The cells on the lower side of the member were fixed and then stained with 10% Giemsa. Cell number that had migrated through the pores was quantified by counting ten independent visual fields under the microscope for statistics.

### Western blot

Total protein from CD133+ SGC-7901 and MKN45 cells was isolated and quantified using RIPA Lysis Buffer and BCA Protein Assay Kit (Beyotime, China) respectively. Each equal amount of protein was run on 10% sodium dodecyl sulfate/polyacrylamide gel electrophoresis (SDS/PAGE), then transferred to PVDF membranes. The membranes were blocked with 5% non-fat milk for 2 h, and the blots were incubated with primary antibody against thymidylate synthase (TS) and cleaved caspase 3 (c-caspase 3) overnight at 4°C, with β-actin acting as control, then incubated with HRP-conjugated secondary antibody (1: 5000 goat anti-rabbit) for 2 h at room temperature. The bands were visualized using BeyoECL Plus ECL Kit (Beyotime, China) and images by gel image analysis system.

### TUNEL assay

The cell apoptosis was assessed using the terminal deoxynucleotide transferase dUTP nick end labeling (TUNEL) staining in accordance with the manufacturer’s instructions. After dehydration by ethanol, TUNEL reaction mixture was added and incubated with cells for 1 h at 37°C. The residual liquid was removed via washing with PBS. The cells were stained using 3,3′-diaminobenzidine (DAB) as a substrate for the peroxidase at room temperature for 10 min. For each section, ten different fields were randomly selected for counting at least 150 cells from at least three separate experiments. The number of TUNEL-positive cells was analyzed using light microscope system at 400× magnification in a blinder manner. Positive apoptotic cells were stained claybank [23].

### ^3^H-Thymidine assays

^3^H-Thymidine was utilized for *in vitro* assessment of thymidine pathway activity in cultured CD133+ SGC-7901 and MKN45 cells. Cells were seeded in six-well plate in RPMI-1640 supplemented with 10% FBS and antibiotics, incubated 24 h in 5% CO_2_ at 37°C. When cell cultures reached 70% confluence, cells were exposed to treatment with either scFV–NPs, scFV–Pemetrexed–NPs, or scFV–METase–Pemetrexed–NPs in growth media. Drug-containing medium was then removed, and the cells were then washed and pulsed with 5 μCi of ^3^H-thymidine per well for 1 h. The cells were then washed and scraped into plastic vials. Scintillant was added to each vial and the radioactivity was counted on a scintillation counter.

### METase activity assay

The METase activities of the CD133+ SGC-7901 and MKN45 cells were measured according to the method of the previous study [24]. Briefly, 1 × 10^7^ cells were collected after trypsin-ethylenediaminetetraacetic acid (EDTA) digestion. Cell pellets were washed with PBS and diluted. The cells were homogenized by sonication for 1 min with centrifugation at 14000 rpm for 10 min. The activity of METase was measured in supernatant by determining α-ketobutyrate production from 10 mM methionine using 3-methyl-2-benzo-thiazoline hydrazone. The concentration of reaction product was measured with a Hitachi model U-2000 spectrophotometer at 335 nm absorbance value. The amount of protein in the cell lysate was determined with the Lowry Reagent Kit using bovine serum albumin as a standard. Specific METase activity was calculated as mU/mg protein.

### Measurement of free methionine levels

The methionine level in the cell lysates was determined by high-performance liquid chromatography after derivatization of amino acids with the fluorescent reagent *o*-phthaldialdehyde (OPA) [25,26]. The cell lysate was first precipitated by acetonitrile to form mixture (1000 ml) in 1:4 volume ratio. Then, partial supernatant (10 ml) was mixed with 5 ml of OPA. After 1 min, 50 ml of sodium acetate (0.1 mM, pH = 7.0) was added, and 20 ml of sample was loaded on a reversed phase Supercosil LC-18-DB (Supelco, Bellefonte, PA, U.S.A.) column at room temperature. The amino acid derivatives were resolved with solution A (tetrahydrofuran/methanol/0.1 M sodium acetate (pH = 7.2): 5/95/900) and solution B (methanol). A gradient from 20 to 60% was run at a flow rate of 1.5 ml per minute. The eluate was read with a fluorescence spectrophotometer at 350–450 nm.

### Statistical analysis

All data were expressed as mean ± standard error (SE). The differences among three or more groups were analyzed with one-way ANOVA, and the differences between two groups was compared with independent-samples *t* test. *P*<0.05 was considered as statistically significant.

## Results

### Structure characterization of METase/Pemetrexed-loaded scFV–NPs and *in vitro* drug release

**Figure 1:**
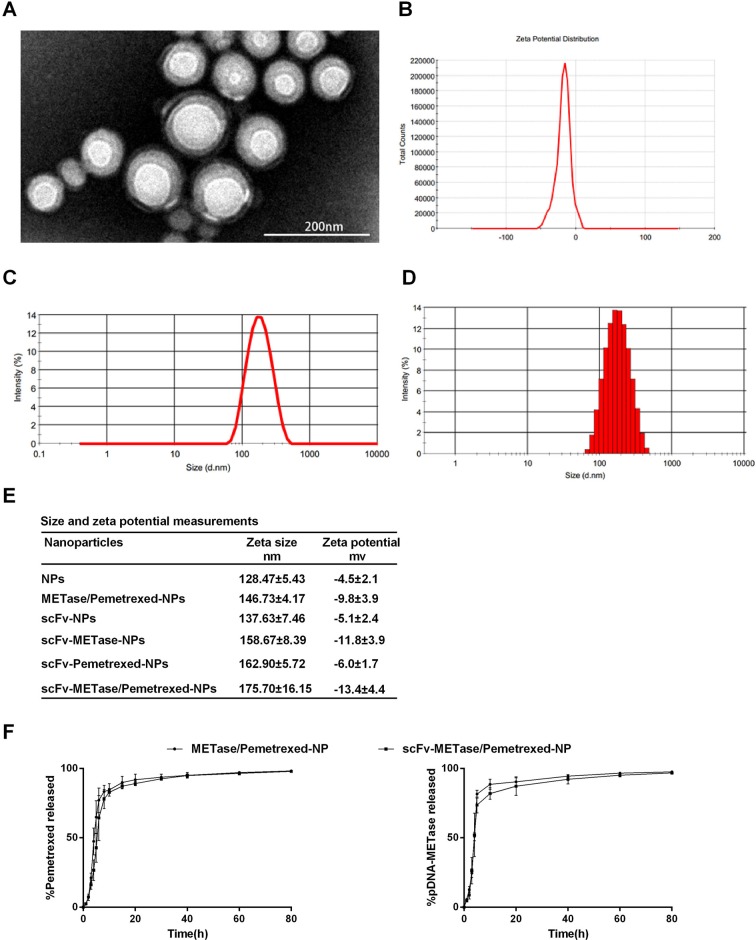
Structure characterization of METase/Pemetrexed-loaded scFV–NPs and *in vitro* drug release (**A**) TEM image of scFV–METase–/Pemetrexed–NPs. (**B**) Zeta potential distribution of scFV–METase–/Pemetrexed–NPs. (**C** and **D**) Size distribution based on intensity of scFV–METase–/Pemetrexed–NPs. (**E**) Size and Zeta potential analysis of different NPs. (**F**) *In vitro* release of pemetrexed and pDNA–METase from METase–/Pemetrexed-loaded-NPs and scFV–METase–/Pemetrexed-loaded-NPs. All data were expressed as mean ± standard error (SE).

TEM imaging of scFV–METase/Pemetrexed–NPs revealed that PEG/PLGA complexes with scFV functionalization are spheres with narrow size distribution and a smooth surface (Figure 1A). The representative graphs of zeta potential distribution and zeta size about scFV–METase/Pemetrexed–NPs were indicated in Figure 1B–D. Owing to the dry conditions observed with TEM, particle size was smaller than that determined via dynamic light scattering in aqueous solution. Size and zeta potential measurements of various NPs were shown in Figure 1E. The average particle zeta size of scFV–NPs was higher than that of NPs without scFV decoration. Moreover, the scFV–METase–Pemetrexed-loaded NPs have larger particle size compared with scFV–METase NPs or scFV–Pemetrexed NPs. Although statistics differences could not be demonstrated, scFV-functionalized NPs had decreased zeta potential, as compared with non-functionalized NPs. The drug release profiles of METase/Pemetrexed–NPs and scFV–METase–Pemetrexed-NPs *in vitro* were shown in Figure 1F. All samples displayed rapid drug release until about 12 h, and followed by relatively slow and steady drug release.

### Cell growth and migration ability of CD133+ gastric cancer cell

**Figure 2:**
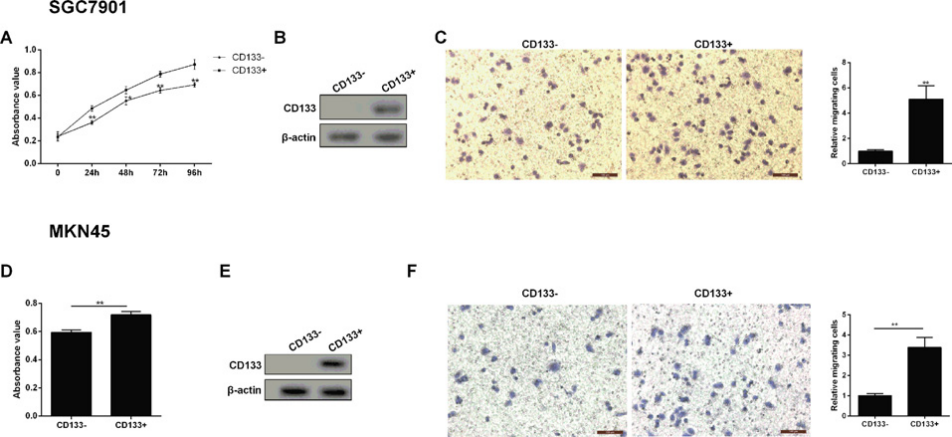
Cell growth and migration ability of CD133+ gastric cancer cell (**A**) Cell growth was detected by MTT method at 0, 24, 48, 72, and 96 h point. (**B**) The expression of CD133 protein was assessed by Western blot in CD133− SGC7901 and CD133+ SGC7901 cells. (**C**) Representative images of gastric cancer cell (SGC7901) migration and quantized histogram of relative migrating SGC7901 cells. (**D**) MKN45 cell growth was detected by MTT method at 0, 24, 48, 72, and 96 h point. (**E**) The expression of CD133 protein was assessed by Western blot in CD133− MKN45 and CD133+ MKN45 cells. (**F**) Representative images of gastric cancer cell (MKN45) migration and quantized histogram of relative migrating MKN45 cells. All data were expressed as mean ± standard error (SE); *P<0.05, **P<0.01 vs CD133− SGC7901 cells. β-Actin was used for internal control in Western blot analysis.

CD133+ SGC7901 and MKN45 gastric cancer cells were isolated, then the absorbance value and migration ability of gastric cancer cells were corresponding to be measured. As shown in Figure 2A and D, the cell viability was significantly increased in the CD133+ gastric cancer cells than in the CD133 negative expression group, and showing a time-dependent manner. Meanwhile, CD133 positive expression gastric cancer cells had an obvious up-regulated CD133 protein level relative to the CD133− group (Figure 2B and E). Transwell assays were conducted to assess the effects of CD133 positive expression on the migration ability of gastric cancer cells. The migration ability of gastric cancer cells was remarkably lower in the CD133− group than in the CD133+ group (Figure 2C and F).

### The effects of scFV–METase–Pemetrexed-loaded NPs on cell growth and METase activity

**Figure 3:**
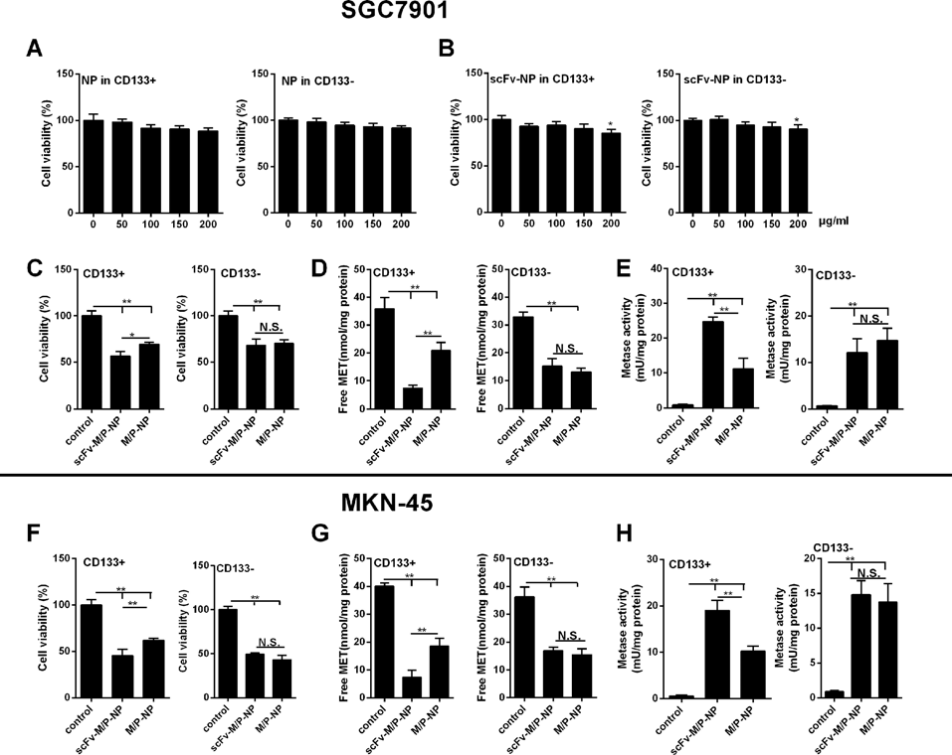
The effects of scFV–METase–Pemetrexed-loaded NPs on cell growth and METase activity (**A**) Cell viability in PEG/PLGA–NPs, also named NPs. (**B**) Cell viability of scFV–NPs under the condition of different transfection amount. (**C** and **F**) Cell viability of control, METase–/Pemetrexed–NPs and scFV–METase–/Pemetrexed–NPs. (**D** and **G**) Free METase levels *in vitro*. (**E** and **H**) METase activity *in vitro*. Methioninase activity was measured with 3-methyl-2-benzothiazoline hydrazone. Cellular free methionine levels were measured by OPA-derivitized amino acids separated by high-performance liquid chromatography (see “Materials and methods” section for a description of procedures). In figure panel (B), *P<0.05 vs scFV–Nps with 0 μg/ml transfection amount. In figure panels (C–F), *P<0.05, **P<0.01 comparison between groups.

CD133+ SGC7901 and MKN45 cells showed prominent cell growth and migration ability; thus, it was chosen for the following experiments. From Figure 3A, it appeared that NPs had no significant toxic effect on CD133+ marked gastric cancer cells (SGC7901). Similarly, there was no significant statistical difference in the SGC7901 cell viability until the transfection amount up to 200 μg/ml (Figure 3B). The similar situation existed in MKN45 cells (the data were not shown). Moreover, further analysis demonstrated that scFV–METase–Pemetrexed NPs or METase–Pemetrexed NPs both showed obvious inhibition effects on cell viability, especially in scFV–METase–Pemetrexed-loaded NPs, when compared with that in the control group (Figure 3C and F). The free MET concentration and METase activity were subsequently detected by corresponding methods. Results found that METase–Pemetrexed-loaded NPs with or without scFV decoration significantly decreased the production of free MET in CD133+ SGC7901 and MKN45 cells, and the reduction trend was more obvious in scFV–METase–Pemetrexed-loaded NPs (Figure 3D and G). On the contrary, the METase activity in scFV–METase–Pemetrexed NPs and METase–Pemetrexed-loaded NPs group was markedly up-regulated, and scFV–METase–Pemetrexed NPs showed stronger METase activity than that in the METase–Pemetrexed-loaded NPs (Figure 3E and H).

### METase enhanced the inhibitory effect of Pemetrexed on thymidylate synthase and cell apoptosis

**Figure 4:**
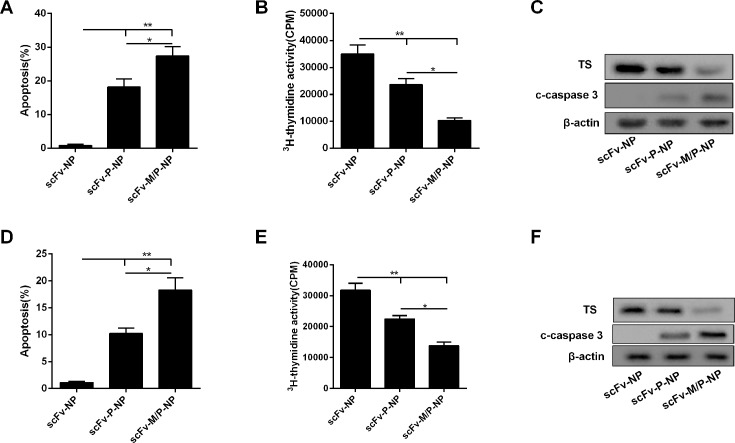
METase enhanced the inhibitory effect of Pemetrexed on thymidylate synthase and cell apoptosis (**A**) Cell apoptosis in CD133+ SGC7901 cells treated with control, METase–/Pemetrexed–NPs and scFV–METase–/Pemetrexed–NPs. (**B**) ^3^H-thymidine assay was performed on CD133+ SGC7901 cells in untreated control (scFV–NPs), pemetrexed (scFV–pemetrexed–NPs), and combination therapy with METase (scFV–METase/pemetrexed–NPs). (**C**) The protein levels of thymidylate synthase (TS) and c-caspase 3 in gastric cancer cells. (**D**) Cell apoptosis in CD133+ MKN45 cells treated with control, METase–/Pemetrexed–NPs and scFV–METase–/Pemetrexed–NPs. (**E**) ^3^H-Thymidine assay was performed on CD133+ MKN45 cells in untreated control (scFV–NPs), pemetrexed (scFV–pemetrexed–NPs), and combination therapy with METase (scFV–METase/pemetrexed–NPs). (**F**) The protein levels of thymidylate synthase (TS) and c-caspase 3 in gastric cancer cells.All data were expressed as mean ± standard error (SE); *P<0.05, **P<0.01 comparison between groups. β-Actin was used for internal control in Western blot.

scFV–METase–Pemetrexed NPs with or without scFV decoration both showed obvious inhibition effects on cell viability. Next, the percentage of cell apoptosis and apoptosis related proteins were assessed. As shown in Figure 4A and D, when compared with scFV–NPs group, scFV–Pemetrexed–NPs and scFV–METase–Pemetrexed–NPs both induced apoptosis, and the percentage of gastric cancer cell apoptosis was significantly higher in scFV–METase–Pemetrexed-loaded NPs group than that of scFV–Pemetrexed NPs. Furthermore, ^3^H-thymidine assays were conducted to assess the effects of METase on the inhibition of Pemetrexed on thymidine synthesis. The ^3^H-thymidine activity in scFV–Pemetrexed–NPs and scFV–METase–Pemetrexed–NPs was observably down-regulated, particularly in scFV–METase–Pemetrexed–NPs, as compared with scFV–NPs group (Figure 4B and E). In addition, the protein levels of c-caspase 3 and TS were detected by Western blot method. scFV–Pemetrexed–NPs promoted the expression of c-caspase 3 while repressed the production of TS protein. scFV–METase–Pemetrexed–NPs enhanced these effects, which showing up-regulated c-caspase 3 protein production and down-regulated TS expression (Figure 4C and F).

## Discussion

Genetic approaches in recent years have presented the significant clinical impact in gastric carcinoma patients [27], while the therapeutic effect is not quiet perfect. At present, anticancer drug NPs preparation has become an important research field of gastric cancer treatment. Nanocarriers for gastric targeted drug delivery system can effectively deliver the drug to the stomach lesion and reduce the potential damage to other organs [8]. Overexpression of CD133 protein is detected in many malignancies including non-small cell lung cancer (NSCLC), brain tumor, liver cancer, and colon cancer [4,28–30]. Recently, it has been reported that CD133 is highly expressed in gastric carcinoma [6]. Similarly, in the present study, we found that the protein level of CD133 was significantly increased in CD133+ gastric cancer cell. Moreover, the growth and migration ability of CD133+ SGC7901 and MKN45 cell were significantly increased compared with those of CD133−cells. All these outcomes indicated that CD133+ has a strong capacity of promoting the proliferation of gastric cancer cell and it's of importance to target this type of cell.

Nowadays, great quantities of targeted anticancer drugs have emerged, also accompanied by high affinity ligand-functionalized polymer NPs for drug delivery [31], and nanocarriers have been widely used in the antitumor drugs deliver system. In particular, biodegradable polymers PEG and PLGA are excellent candidates with good biocompatible and immunogenicity, and the application of diblock copolymers has demonstrated enormous potential as drug delivery carriers [32]. Furthermore, scFv is also used to enhance targeting effects and promote the effective delivery of anticancer drugs [33]. Here, in the present study, the PEG–PLGA nanocarriers modified with anti-CD133–scFV are constructed successfully and are used for the following experimental procedures.

Pemetrexed, acted as one kind of antitumor drugs, is receiving more and more attention in the treatment of gastric carcinoma. Celio et al. [34] reported that pemetrexed in combination with oxaliplatin could be used as a first-line therapy for advanced gastric cancer (AGC). Pemetrexed in combination with cisplatin is effective and safe for AGC patients [35]. In the present study, two therapeutic agents including METase and pemetrexed were combined and coencapsulated into PEG–PLGA NPs, and the biological functions of CD133+ SGC7901 and MKN45 cells were investigated. Results indicated that the cell viability and free METase concentration were reduced, while the activity of METase was up-regulated in NPs carrying pemetrexed. At the same time, scFV–NPs decorated with pemetrexed evidently enhanced cell apoptosis, showing the increased number of apoptosis and c-caspase 3 protein level; meantime, it observably inhibited DNA synthesis, presenting the decreased ^3^H-thymidine assays and DNA synthesis key enzyme (TS) expression. Thus, we can conclude that pemetrexed has antitumor efficacy and the potential of the pemetrexed as a therapeutic strategy was preliminary explored.

It has been believed that enhanced requirement of METase is necessary for cancer cells, as compared with normal cells. Methionine dependence may be the only known general and common metabolic defect in cancers [36]. Moreover, previous studies indicated METase is a specific tumor-targeting agent that may be applicable to the treatment of multiple types of cancers [36–40]. Through the research, we found that METase-loaded NPs with or without scFV decoration significantly inhibited CD133+ SGC7901 cell viability and reduced free MET concentration, also increased Metase activity; meanwhile, scFV NPs modified with METase can remarkably promote cell apoptosis and c-caspase 3 protein expression, also repress ^3^H-thymidine assays and TS enzyme level. More significantly, METase enhanced the inhibitory effects of pemetrexed on TS synthesis and cell apoptosis. Therefore, METase therapy has many powerful features and appears to have potential as a therapeutic agent for gastric cancer treatment.

## Conclusion

PEG–PLGA NPs modified with anti-CD133–scFV for drug delivery were successfully prepared. METase and pemetrexed were enclosed within scFV–PEG–PLGA NPs with small particle size. These functional scFV– PEG–PLGA NPs exhibited lower cell cytoxicity and increased apoptosis in CD133+ SGC-7901 cells. Increased therapeutic efficiency of scFV–METase/pemetrexed–NPs was observed in CD133+ SGC-7901 cells *in vitro*, compared with scFV–pemetrexed–NPs. Thus, our findings demonstrated a new strategy of combination tumor therapy with the METase and pemetrexed, and further *in vivo* studies are being directed toward enhancement of the anticancer effectiveness of METase–pemetrexed.

## Abbreviations

AGC: advanced gastric cancer
c-caspase 3: cleaved caspase 3
DCM: dichloromethane
DLS: dynamic light scattering
MACS: magnetic-activated cell sorting
METase: methioninase
NHS: *N*-hydroxy succinimide
NP: nanoparticle
OPA: *o*-phthaldialdehyde
PEG: polyethylene glycol
PLGA: poly(D,L-lactide-co-glycolide acid)
PVA: polyvinyl alcohol
scFv: single-chain variable fragment
TPN: total parenteral nutrition

## References

[1] Xu W., Chen Q., Wang Q., Sun Y., Wang S., Li A. (2014) JWA reverses cisplatin resistance via the CK2—XRCC1 pathway in human gastric cancer cells. Cell Death Dis. 5, e1551 10.1038/cddis.2014.517 25476899 PMC4649833

[2] Zhao P., Li Y. and Lu Y. (2010) Aberrant expression of CD133 protein correlates with Ki-67 expression and is a prognostic marker in gastric adenocarcinoma. BMC Cancer 10, 1–6 10.1186/1471-2407-10-218 20487522 PMC2891633

[3] Quirici N., Soligo D., Caneva L., Servida F., Bossolasco P. and Deliliers G.L. (2001) Differentiation and expansion of endothelial cells from human bone marrow CD133+ cells. Br. J. Haematol. 115, 186–194 10.1046/j.1365-2141.2001.03077.x 11722432

[4] Riccivitiani L., Lombardi D.G., Pilozzi E., Biffoni M., Todaro M., Peschle C. (2007) Identification and expansion of human colon-cancer-initiating cells. Nature 445, 111 10.1038/nature05384 17122771

[5] Liu G., Yuan X., Zeng Z., Tunici P., Ng H., Abdulkadir I.R. (2006) Analysis of gene expression and chemoresistance of CD133 + cancer stem cells in glioblastoma. Mol. Cancer 5, 67 10.1186/1476-4598-5-67 17140455 PMC1697823

[6] Smith L.M., Nesterova A., Ryan M.C., Duniho S., Jonas M., Anderson M. (2008) CD133/prominin-1 is a potential therapeutic target for antibody-drug conjugates in hepatocellular and gastric cancers. Br. J. Cancer 99, 100 10.1038/sj.bjc.6604437 18542072 PMC2453027

[7] Liang X., Li X., Chang J., Duan Y. and Li Z. (2013) Properties and evaluation of quaternized chitosan/lipid cation polymeric liposomes for cancer-targeted gene delivery. Langmuir 29, 8683–8693 10.1021/la401166v 23763489

[8] Thi T.D.L., Pham T.H., Nghia Nguyen T., Giang Ngo T.H., Nhung Hoang T.M. and Huan Le Q. (2016) Evaluation of anti-HER2 scFv-conjugated PLGA-PEG nanoparticles on 3D tumor spheroids of BT474 and HCT116 cancer cells. Adv. Nat. Sci. Nanosci. Nanotechnol. 7, 025004 10.1088/2043-6262/7/2/025004

[9] Siegel H.K.M. and Steven J. (2011) Poly lactic-co-glycolic acid (PLGA) as Biodegradable controlled drug delivery carrier. Polymers 3, 1377 10.3390/polym3031377 22577513 PMC3347861

[10] Jeong B., You H.B., Lee D.S. and Kim S.W. (1997) Biodegradable block copolymers as injectable drug-delivery systems. Nature 388, 860–862 10.1038/42218 9278046

[11] Chang W.K., Tai Y.J., Chiang C.H., Hu C.S., Hong P.D. and Yeh M.K. (2011) The comparison of protein-entrapped liposomes and lipoparticles: preparation, characterization, and efficacy of cellular uptake. Int. J. Nanomed. 6, 2403–2417 22072876 10.2147/IJN.S25646 PMC3205135

[12] Zhao H. and Lin Y.L.Y. (2008) Selectivity of folate conjugated polymer micelles against different tumor cells. Int. J. Pharm. 349, 256–268 10.1016/j.ijpharm.2007.07.040 17850996

[13] Xin L., Cao J., Cheng H., Zeng F. and Hu X. (2013) Stealth cationic liposomes modified with anti-CAGE single-chain fragment variable deliver recombinant methioninase for gastric carcinoma therapy. J. Nanosci. Nanotechnol. 13, 178–183 10.1166/jnn.2013.6881 23646714

[14] Song Y.Q. and Wen-Ping L. (2004) Cost-effective comparison between early enteral nutrition and total parenteral nutrition on elder postoperative gastric cancer patients. Chinese J. Gen. Surg., 19, 97–99

[15] Kim Y., Chung H., Kang W.k., Park S., Kim C., Kim T. (2008) Pemetrexed and cisplatin in patients with advanced gastric cancer: a Korean cancer study group multicenter phase II study. Cancer Chemother. Pharmacol. 62, 263–270 10.1007/s00280-007-0600-y 17960386

[16] Kim J.H., Lee K.W., Jung Y., Tai Y.K., Ham H.S., Jong H.S. (2005) Cytotoxic effects of pemetrexed in gastric cancer cells. Cancer Sci. 96, 365–371 10.1111/j.1349-7006.2005.00058.x 15958060 PMC11160028

[17] Wei G.L., Huang X.E., Huo J.G., Wang X.N. and Tang J.H. (2013) Phase II study on pemetrexed-based chemotherapy in treating patients with metastatic gastric cancer not responding to prior palliative chemotherapy. Asian Pac. J. Cancer Prev. 14, 2703–2706 10.7314/APJCP.2013.14.5.2703 23803018

[18] Liu J., Huang X.E. and Feng J.F. (2014) Further study on pemetrexed based chemotherapy in treating patients with advanced gastric cancer (AGC). Asian Pac. J. Cancer Prev. 15, 6587–6590 10.7314/APJCP.2014.15.16.6587 25169492

[19] Zhu X., Bidlingmaier S., Hashizume R., James C.D., Berger M.S. and Liu B. (2010) Identification of internalizing human single-chain antibodies targeting brain tumor sphere cells. Mol. Cancer Ther. 9, 2131 10.1158/1535-7163.MCT-09-1059 20587664 PMC2944778

[20] Lin W.J., Lee W.C. and Shieh M.J. (2017) Hyaluronic acid conjugated micelles possessing CD44 targeting potential for gene delivery. Carbohydr. Polym. 155, 101–108 10.1016/j.carbpol.2016.08.021 27702492

[21] Haggag Y., Abdelwahab Y., Ojo O., Osman M., Elgizawy S., Eltanani M. (2015) Preparation and in vivo evaluation of insulin-loaded biodegradable nanoparticles prepared from diblock copolymers of PLGA and PEG. Int. J. Pharm. 499, 236–246 10.1016/j.ijpharm.2015.12.063 26746800

[22] Xin L., Caot J.Q., Liu C., Zeng F., Cheng H., Hu X.Y. (2015) Evaluation of rMETase-loaded stealth PLGA/liposomes modified with anti-CAGE scFV for treatment of gastric carcinoma. J. Biomed. Nanotechnol. 11, 1153 10.1166/jbn.2015.2062 26307838

[23] Wang S., Zhong Z., Wan J., Tan W., Wu G., Chen M. (2013) Oridonin induces apoptosis, inhibits migration and invasion on highly-metastatic human breast cancer cells. Am. J. Chin. Med. 41, 177–196 10.1142/S0192415X13500134 23336515

[24] Tanaka H., Esaki N. and Soda K. (1977) Properties of L-methionine gamma-lyase from Pseudomonas ovalis. Biochemistry 16, 100 10.1021/bi00620a016 831771

[25] Tan Y., Sun X., Xu M., An Z., Tan X., Han Q. (1998) Polyethylene glycol conjugation of recombinant methioninase for cancer therapy. Protein Expr. Purif. 12, 45–52 10.1006/prep.1997.0805 9473456

[26] Miki K., Xu M., An Z., Wang X., Yang M., Al R.W. (2000) Survival efficacy of the combination of the methioninase gene and methioninase in a lung cancer orthotopic model. Cancer Gene Ther. 7, 332–338 10.1038/sj.cgt.7700103 10770644

[27] Zhou J., Shen J., Seifer B.J., Jiang S., Wang J., Xiong H. (2016) Approaches and genetic determinants in predicting response to neoadjuvant chemotherapy in locally advanced gastric cancer. Oncotarget 8, 10.18632/oncotarget.12955 PMC5444758 27802185

[28] Hilbe W., Dirnhofer S., Oberwasserlechner F., Schmid T., Gunsilius E., Hilbe G. (2004) CD133 positive endothelial progenitor cells contribute to the tumour vasculature in non-small cell lung cancer. J. Clin. Pathol. 57, 965 10.1136/jcp.2004.016444 15333659 PMC1770433

[29] Singh S., Hawkins C., Clarke I., Squire J., Bayani J., Hide T. (2004) Identification of human brain tumour initiating cells. Nature 432, 396–401 10.1038/nature03128 15549107

[30] Zhou L., Wei X., Cheng L., Tian J. and Jiang J.J. (2007) CD133, one of the markers of cancer stem cells in Hep-2 cell line. Laryngoscope 117, 455–460 10.1097/01.mlg.0000251586.15299.35 17334305

[31] Sadat S.M., Saeidnia S., Nazarali A.J. and Haddadi A. (2015) Nano-pharmaceutical formulations for targeted drug delivery against HER2 in breast cancer. Curr. Cancer Drug Targets 15, 71 10.2174/1568009615666150105115047 25564255

[32] Rafiei P. and Haddadi A. (2017) Docetaxel-loaded PLGA and PLGA-PEG nanoparticles for intravenous application: pharmacokinetics and biodistribution profile. Int. J. Nanomed. 12, 935–947 10.2147/IJN.S121881 28184163 PMC5291330

[33] Weisser N.E. and Hall J.C. (2009) Applications of single-chain variable fragment antibodies in therapeutics and diagnostics. Biotechnol. Adv. 27, 502–520 10.1016/j.biotechadv.2009.04.004 19374944

[34] Celio L., Sternberg C.N., Labianca R., Torre I.L., Amoroso V., Barone C. (2009) Pemetrexed in combination with oxaliplatin as a first-line therapy for advanced gastric cancer: a multi-institutional phase II study. Ann. Oncol. 20, 1062–1067 10.1093/annonc/mdn766 19218305

[35] Bang Y., Kim Y., Chung H.C., Kang W., Park S., Yang S. (2006) A multicenter phase II study of pemetrexed and cisplatin in patients with advanced gastric cancer (AGC). J. Clin. Oncol., 62, 10.1007/s00280-007-0600-y 17960386

[36] Hoffman R.M. (2014) Development of recombinant methioninase to target the general cancer-specific metabolic defect of methionine dependence: a 40-year odyssey. Expert Opin. Biol. Ther. 15, 21–31 10.1517/14712598.2015.963050 25439528

[37] Tan Y., Xu M. and Hoffman R.M. (2010) Broad selective efficacy of recombinant methioninase and polyethylene glycol-modified recombinant methioninase on cancer cells in vitro. Anticancer Res. 30, 1041–1046 20530407

[38] Yano S., Takehara K., Ming Z., Tan Y., Han Q., Li S. (2016) Tumor-specific cell-cycle decoy by Salmonella typhimurium A1-R combined with tumor-selective cell-cycle trap by methioninase overcome tumor intrinsic chemoresistance as visualized by FUCCI imaging. Cell Cycle, 15, 1715–1723 10.1080/15384101.2016.1181240 27152859 PMC4957602

[39] Yano S., Li S., Han Q., Tan Y., Bouvet M., Fujiwara T. (2014) Selective methioninase-induced trap of cancer cells in S/G2 phase visualized by FUCCI imaging confers chemosensitivity. Oncotarget 5, 8729–8736 10.18632/oncotarget.2369 25238266 PMC4226717

[40] Yano S., Zhang Y., Zhao M., Hiroshima Y., Miwa S., Uehara F. (2011) Tumor-targeting Salmonella typhimurium A1-R decoys quiescent cancer cells to cycle as visualized by FUCCI imaging and become sensitive to chemotherapy. Cell Cycle 13, 3958–3963 10.4161/15384101.2014.964115 PMC4615054 25483077

