# Correction: Evaluation of METase-pemetrexed-loaded PEG-PLGA nanoparticles modified with anti-CD133-scFV for treatment of gastric carcinoma

**DOI:** 10.1042/BSR-20171001_COR

**Published:** 2020-12-16

**Keywords:** CD133, gastric carcinoma, methioninase, PEG-PLGA nanoparticles, pemetrexed, scFV

The authors of the original article “Evaluation of METase-Pemetrexed-Loaded PEG-PLGA Nanoparticles Modified with Anti-CD133-scFV for Treatment of Gastric Carcinoma” (Biosci Rep (2018) **38**(1), DOI: 10.1042/BSR20171001) would like to correct Figure 2C. During the authors' figure build of Figure 2C, the migration images in the “CD133-” group and the “CD133+” group had been switched. The authors declare that these corrections do not change the results or conclusions of their paper, and would like to apologize for any inconvenience caused. All of the authors have approved the correction. The correct version of Figure 2 is presented here.

**Figure 2:**
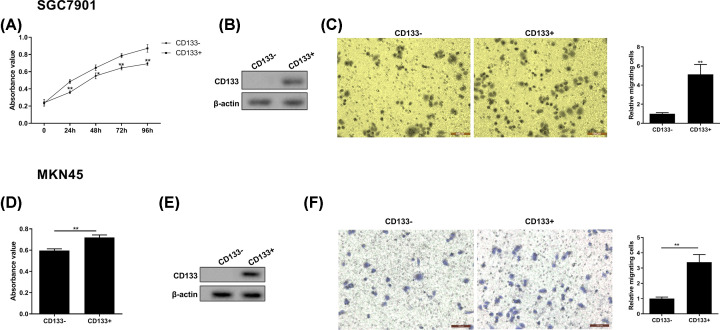
Cell growth and migration ability of CD133+ gastric cancer cell (**A**) Cell growth was detected by MTT method at 0, 24, 48, 72, and 96 h point. (**B**) The expression of CD133 protein was assessed by Western blot in CD133− SGC7901 and CD133+ SGC7901 cells. (**C**) Representative images of gastric cancer cell (SGC7901) migration and quantized histogram of relative migrating SGC7901 cells. (**D**) MKN45 cell growth was detected by MTT method at 0, 24, 48, 72, and 96 h point. (**E**) The expression of CD133 protein was assessed by Western blot in CD133− MKN45 and CD133+ MKN45 cells. (**F**) Representative images of gastric cancer cell (MKN45) migration and quantized histogram of relative migrating MKN45 cells. All data were expressed as mean ± standard error (SE); *P<0.05, **P<0.01 vs CD133− SGC7901 cells. β-Actin was used for internal control in Western blot analysis.

