# Expression of concern: evaluation of METase-pemetrexed-loaded PEG-PLGA nanoparticles modified with anti-CD133-scFV for treatment of gastric carcinoma

**DOI:** 10.1042/BSR-20171001_EOC

**Published:** 2021-02-16

**Keywords:** CD133, gastric carcinoma, methioninase, PEG-PLGA nanoparticles, pemetrexed, scFV

The Editorial Office has been made aware of potential issues surrounding the scientific validity of this paper, hence has issued an expression of concern to notify readers whilst the Editorial Office investigates.

